# Diagnostic Performance of the Magnetic Resonance Parkinsonism Index in Differentiating Progressive Supranuclear Palsy from Parkinson’s Disease: An Updated Systematic Review and Meta-Analysis

**DOI:** 10.3390/diagnostics12010012

**Published:** 2021-12-22

**Authors:** Seongken Kim, Chong Hyun Suh, Woo Hyun Shim, Sang Joon Kim

**Affiliations:** Department of Radiology and Research Institute of Radiology, Asan Medical Center, University of Ulsan College of Medicine, Seoul 05505, Korea; closea@naver.com (S.K.); ssimu81@gmail.com (W.H.S.); sjkimjb@amc.seoul.kr (S.J.K.)

**Keywords:** progressive supranuclear palsy, Parkinson’s disease, magnetic resonance parkinsonism index, meta-analysis

## Abstract

Progressive supranuclear palsy (PSP) and Parkinson’s disease (PD) are difficult to differentiate especially in the early stages. We aimed to investigate the diagnostic performance of the magnetic resonance parkinsonism index (MRPI) in differentiating PSP from PD. A systematic literature search of PubMed-MEDLINE and EMBASE was performed to identify original articles evaluating the diagnostic performance of the MRPI in differentiating PSP from PD published up to 20 February 2021. The pooled sensitivity, specificity, and 95% CI were calculated using the bivariate random-effects model. The area under the curve (AUC) was calculated using a hierarchical summary receiver operating characteristic (HSROC) model. Meta-regression was performed to explain the effects of heterogeneity. A total of 14 original articles involving 484 PSP patients and 1243 PD patients were included. In all studies, T1-weighted images were used to calculate the MRPI. Among the 14 studies, nine studies used 3D T1-weighted images. The pooled sensitivity and specificity for the diagnostic performance of the MRPI in differentiating PSP from PD were 96% (95% CI, 87–99%) and 98% (95% CI, 91–100%), respectively. The area under the HSROC curve was 0.99 (95% CI, 0.98–1.00). Heterogeneity was present (sensitivity: I^2^ = 97.29%; specificity: I^2^ = 98.82%). Meta-regression showed the association of the magnet field strength with heterogeneity. Studies using 3 T MRI showed significantly higher sensitivity (100%) and specificity (100%) than those of studies using 1.5 T MRI (sensitivity of 98% and specificity of 97%) (*p* < 0.01). Thus, the MRPI could accurately differentiate PSP from PD and support the implementation of appropriate management strategies for patients with PSP.

## 1. Introduction

Progressive supranuclear palsy (PSP) and Parkinson’s disease (PD) are difficult to differentiate especially in the early stages because supranuclear vertical gaze palsy, a characteristic symptom of PSP, does not appear in the early stages of the disease [1,2,3]. In addition, as the supranuclear vertical gaze palsy is characteristic for the most common phenotype of PSP, Richardson’s syndrome (PSP-RS), it may remain absent in PSP-Parkinsonism Predominant (PSP-P) [4,5,6,7]. Magnetic resonance imaging (MRI) has been widely used to differentiate PSP from PD. Various studies have been conducted using MRI with various protocols, including conventional MRI, diffusion-weighted MRI [8,9], susceptibility-weighted MRI [10], and functional MRI [11].

It has been proven that quantitative measurement with conventional MRI is a useful method to differentiate PSP from PD. Various quantitative measures, including the midbrain to pons ratio [12], area of the midbrain [13], volume of the superior cerebellar peduncle [14], and magnetic resonance parkinsonism index (MRPI), have been evaluated. Among the quantitative measures, the MRPI (the pons area to midbrain area ratio multiplied by the middle cerebellar peduncle width to superior cerebellar peduncle width ratio) has been found to accurately differentiate PSP from PD in several articles [15,16,17,18,19,20,21]. Moreover, MRPI in the early stages may be more beneficial in the differential diagnosis of PSP-P, regarding the fact that many other neuroimaging methods as perfusion SPECT do not provide sufficient differentiating [22]. For this reason, several studies on the MRPI have begun to report results according to the PSP phenotype [15,19,23]. A meta-analysis by Zhang et al. [24] reported a pooled sensitivity and specificity of 98% and 99%, respectively, for the MRPI. However, the accuracy of this result is compromised because the authors searched for articles only from PubMed, control groups were heterogeneous, they did not perform meta-regression analysis, and they did not use a hierarchical summary receiver operating characteristic curve (HSROC) model.

Therefore, we aimed to perform an updated systematic review and meta-analysis in terms of the diagnostic performance of the MRPI for the differentiation of PSP from PD.

## 2. Materials and Methods

This study was performed according to the Preferred Reporting Items for Systematic Reviews and Meta-Analyses (PRISMA) guidelines [25].

### 2.1. Literature Search

A systematic literature search of PubMed-MEDLINE and EMBASE was performed to identify original articles evaluating the diagnostic performance of the MRPI for the differentiation of PSP from PD published up to February 20, 2021. The search terms were as follows: ((“progressive supranuclear palsy “) OR (PSP)) AND ((Parkinson disease) OR (parkinsonism)) AND ((“magnetic resonance imaging”) OR (“MR imaging”) OR (“MRI”)). No additional filters were applied.

### 2.2. Eligibility Criteria

To investigate the diagnostic performance of the MRPI for the differentiation of PSP from PD, studies were included if all of the following criteria were met: (1) patients with PSP or PD; (2) patients assessed with the MRPI using T1-weighted MR images; (3) reference standard: clinical diagnosis based on the criteria of each disease, e.g., PD [26,27] or PSP [3,28]; and (4) sufficient information for the reconstruction of 2 × 2 tables to investigate the diagnostic performance of the MRPI for the differentiation of PSP from PD.

Studies were excluded if they were (1) review articles; (2) case reports or case series with less than 10 patients; (3) conference abstracts; (4) editorials, chapters, and notes; (5) studies with a partially overlapping cohort; or (6) studies with incomplete data for the reconstruction of 2 × 2 tables. For studies with a partially overlapping cohort, those with the largest population were selected.

### 2.3. Data Extraction and Quality Assessment

A standardized form was used to extract the following information from the selected studies: (1) Study characteristics: author, institution, duration of patient recruitment, study design, consecutive or non-consecutive enrollment, and reference standard; (2) Demographic and clinical characteristics: total number of patients with PSP or PD, number of patients with PSP, mean age with standard deviation (SD), and male to female ratio; (3) Technical characteristics of MRI: magnetic field strength, vendor, scanner, MR sequences, and number and experience of the reader(s).

Quality assessment of the selected studies was performed using the Quality Assessment of Diagnostic Accuracy Studies-2 (QUADAS-2) tool [29]. The literature search, selection based on eligibility criteria, data extraction, and quality assessment were independently conducted by two reviewers (S.K. and C.H.S.; 2 and 10 years of experience in diagnostic radiology, respectively).

### 2.4. Data Synthesis and Analysis

For each study, we reconstructed 2 × 2 tables. The primary outcome of our study was the diagnostic performance of the MRPI for the differentiation of PSP from PD. To evaluate the diagnostic performance of the MRPI, the pooled sensitivity, specificity, and 95% CI were calculated using the bivariate random-effects and HSROC models, and forest plots were constructed [30,31,32,33]. A HSROC curve with 95% confidence and prediction regions was also plotted.

To assess the heterogeneity among the studies, we used the following tests: (1) Cochran’s Q test with *p* < 0.05 indicating the presence of heterogeneity; (2) Higgins inconsistency index (I^2^) test with a value >50% indicating the presence of heterogeneity [33,34]; (3) Visual assessment of the difference between the 95% confidence region and prediction region in the HSROC curve (large difference indicating heterogeneity); (4) Visual assessment of the coupled forest plots to assess the presence of a threshold effect, i.e., a positive correlation between sensitivity and false positive rate among the selected studies; (5) Spearman correlation coefficient analysis with a value >0.6 revealing a threshold effect [35]. Publication bias was evaluated using Deeks’ funnel plot, and statistical significance was assessed using Deeks’ asymmetry test [36,37]. Meta-regression analysis was performed to explain the effects of heterogeneity. The magnet field strength (1.5 T vs. 3 T) was considered for the bivariate meta-regression model.

Statistical analyses were conducted by one of the authors (C.H.S.; 7 years of experience in performing systematic reviews and meta-analyses) using the “metandi” and “midas” modules in Stata 15.0 (StataCorp, College Station, TX, USA) and the “meta” package in R version 3.1.2 (R Foundation for Statistical Computing, Vienna, Austria). A value of *P* < 0.05 was taken to indicate statistical significance.

## 3. Results

### 3.1. Literature Search

**Figure 1:**
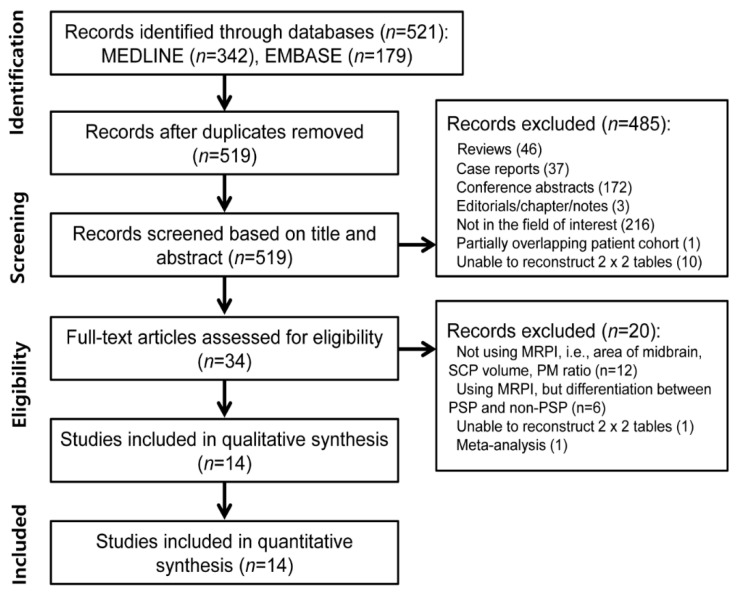
Flow diagram showing the study selection process for systematic review and meta-analysis. MRPI, magnetic resonance parkinsonism index; SCP, superior cerebellar peduncle; PM, pons-midbrain; PSP, progressive supranuclear palsy.

A flowchart of the study selection process is shown in Figure 1. The systematic search identified 521 articles. After the removal of two duplicates, the screening of the titles and abstracts of the 519 remaining articles was performed, and the following 485 articles were excluded: 46 reviews, 37 case reports, 172 conference abstracts, three editorial/chapter/note, 216 articles that were not in the field of interest, one article with a partially overlapping cohort and 10 articles for which the reconstruction of 2 × 2 tables was not possible. A total of 34 full-text articles were further assessed for eligibility, and the following 20 articles were excluded: 12 articles that did not differentiate PSP from PD [12,13,14,38,39,40,41,42,43,44,45,46], six articles that used the MRPI but differentiated between PSP and non-PSP [47,48,49,50,51,52], one article for which the reconstruction of a 2 × 2 table was not possible [53], and one article that was a meta-analysis [24]. Finally, 14 original articles involving 484 PSP patients and 1243 PD patients were included in our meta-analysis [15,16,17,18,19,20,21,23,54,55,56,57,58,59].

### 3.2. Characteristics of the Included Studies

The study and patient characteristics are shown in Table 1. Among the 14 selected studies, two studies were prospective [20,54], eight studies were retrospective [15,17,18,23,56,57,58,59], and four studies did not report their design [16,19,21,55]. Consecutive enrollment was performed in nine studies [15,18,19,20,21,23,54,58,59]; however, five studies did not provide detailed information about patient enrollment [16,17,55,56,57].

The MRI characteristics of the selected articles are shown in Table 2. As Nigro et al. [17] used both 1.5 T and 3 T scanners and obtained the MRPI both manually and automatically, we selected the data for the MRPI obtained manually on 3 T scanners. Of the 14 selected studies, three studies used 3 T scanners [17,19,57], five studies used 1.5 T scanners [15,16,20,55,59], and six studies used 1.5 T or 3 T scanners [18,21,23,54,56,58]. In all studies, T1-weighted images were used to calculate the MRPI; nine studies used 3D T1-weighted images [15,17,19,23,55,56,57,58,59], four studies used conventional T1-weighted images [16,18,20,21], and one study used 3D or conventional T1-weighted images [54]. However, in addition to conventional T1-weighted images, T2-weighted and fluid-attenuated inversion recovery (FLAIR) images were used in one study [18]. The number of readers ranged from 1 to 3; however, one study did not report the number of readers [55]. The section thickness of T1-weighted images ranged from 1 mm to 5 mm in 9 studies [16,17,18,19,20,54,55,56,57], and five studies did not provide information about section thickness [15,21,23,58,59]. The MRPI was manually measured in 12 studies [15,16,17,18,20,21,23,54,55,56,57,59], and automatic measurement of the MRPI was performed in two studies [19,58]. As three studies [19,23,58] measured a new index termed MRPI 2.0, which includes the third and lateral ventricle width in addition to MRPI (MRPI × third ventricle width/frontal horn width), only MRPI data were selected.

### 3.3. Quality Assessment

**Figure 2:**
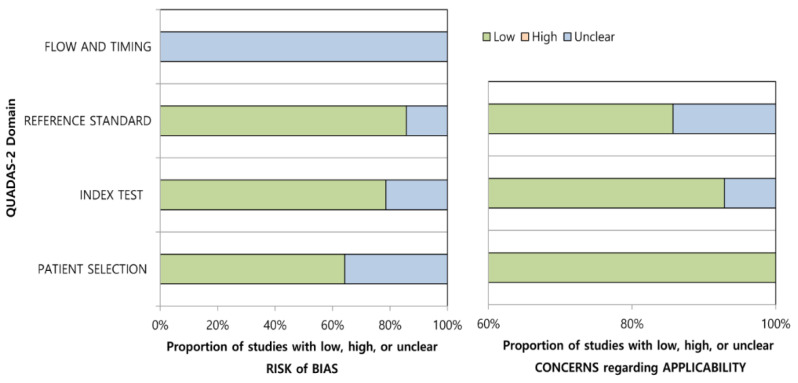
Quality Assessment of Diagnostic Accuracy Studies-2 (QUADAS-2) criteria for the assessment of the 14 included studies.

The results of quality assessment based on QUADAS-2 criteria are shown in Figure 2. Overall, the quality of the studies was considered high. In the patient selection domain, five studies indicated an unclear risk of bias because of their non-consecutive enrollment [16,17,55,56,57]. The remaining studies indicated a low risk of bias [15,18,19,20,21,23,54,58,59], and all of the included studies indicated a low concern on applicability [15,16,17,18,19,20,21,23,54,55,56,57,58,59]. In the index test domain, three studies indicated an unclear risk of bias because it was unclear whether the MRPI was calculated blinded to the reference standard [54,55,58]. There was one study that indicated an unclear concern on applicability because the MRI protocols used for calculating the MRPI were different from those used in other studies [18]. In the reference standard domain, two studies indicated an unclear risk of bias and unclear concern on applicability because of the lack of sufficient information about the diagnosis of PSP or PD [55,57]. In the flow and timing domain, all of the included studies indicated an unclear risk of bias because there was no information about the interval between the index test and reference standard [15,16,17,18,19,20,21,23,54,55,56,57,58,59].

### 3.4. Diagnostic Performance of the MRPI

The sensitivity and specificity of the MRPI in differentiating PSP from PD were available in all 14 studies. The sensitivity and specificity of the studies ranged from 66% to 100% and 68% to 100%, respectively. The cut-off value for the MRPI ranged from 8.98 to 19.42. In addition, the sensitivity and specificity of the studies using 1.5 T scanners ranged from 82% to 100% and 76% to 100%, respectively [15,16,20,55,59]. The sensitivity and specificity of the studies using 3 T scanners ranged from 78% to 100% and 82% to 100%, respectively [17,19,57]. The cut-off value for the MRPI using 1.5 T and 3 T scanners ranged from 10.67 to 19.42 and 13.37 to 13.88, respectively.

**Figure 3:**
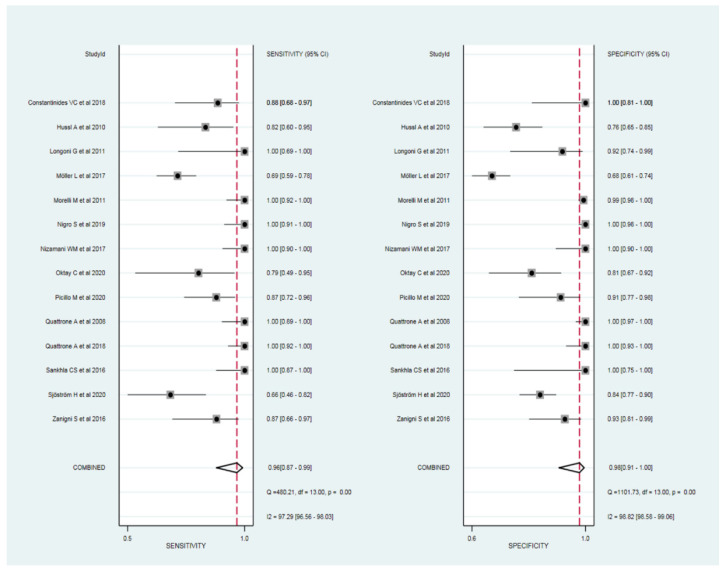
Coupled forest plots of the pooled sensitivity and specificity for the diagnostic performance of the MRPI for the differentiation of progressive supranuclear palsy from Parkinson’s disease. Numbers are pooled estimates with 95% CIs. Horizontal lines represent 95% CIs. Red dotted lines represent pooled sensitivity and specificity, respectively. COMBINED column represent 95% CIs of the pooled sensitivity and specificity. MRPI, magnetic resonance parkinsonism index; CI, confidence interval.

**Figure 4:**
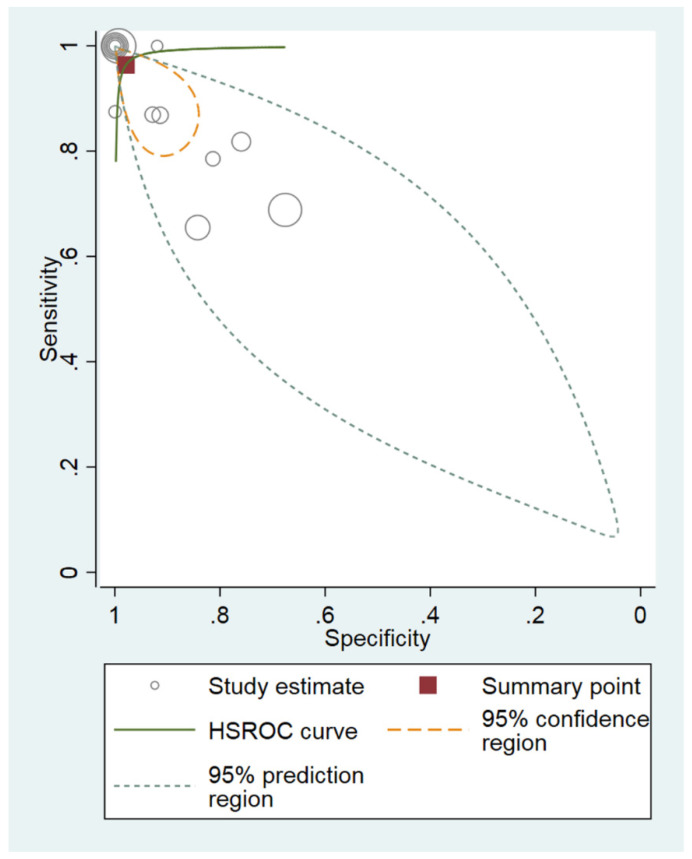
Hierarchical summary receiver operating characteristic (HSROC) curve of the diagnostic performance of the MRPI for the differentiation of progressive supranuclear palsy from Parkinson’s disease. A significant difference was observed between the 95% prediction region and the 95% confidence region, indicating a high possibility of heterogeneity. MRPI, magnetic resonance parkinsonism index.

The pooled sensitivity and specificity for the diagnostic performance of the MRPI in differentiating PSP from PD were 96% (95% CI, 87–99%) and 98% (95% CI, 91–100%), respectively (Figure 3). The area under the HSROC curve was 0.99 (95% CI, 0.98–1.00), which indicated high diagnostic performance (Figure 4).

**Figure 5:**
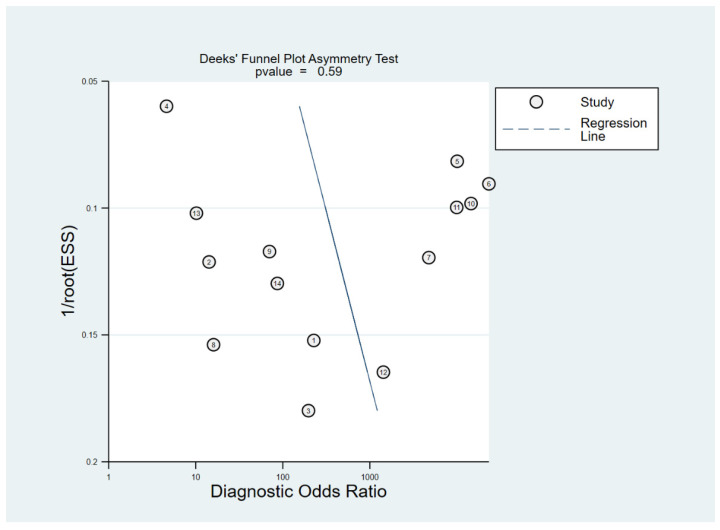
Deek’s funnel plot for the evaluation of potential publication bias. The possibility of publication bias was low.

Cochran’s *Q* test showed that heterogeneity was present among the selected studies (sensitivity: *Q* = 480.21, *p* < 0.01; specificity: *Q* = 1101.73, *p* < 0.01). In addition, Higgins *I*^2^ test showed that heterogeneity was present (sensitivity: *I*^2^ = 97.29%; specificity: *I*^2^ = 98.82%). There was a large difference between the 95% prediction region and the 95% confidence region, indicating a high possibility of heterogeneity among the selected studies. The coupled forest plots indicated no threshold effect, and the Spearman correlation coefficient between sensitivity and false positive rate was −0.841 (95% CI, −0.562–−0.949), also indicating a low likelihood of a threshold effect. Deeks’ funnel plot showed a low possibility of publication bias (*p* = 0.59) (Figure 5).

### 3.5. Meta-Regression

Meta-regression revealed that the magnet field strength was associated with heterogeneity. Although the difference was minimal, studies using 3 T MRI showed significantly higher sensitivity (100%; 95% CI, 98–100%) and specificity (100%; 95% CI, 98–100%) than those of studies using 1.5 T MRI (sensitivity of 98% [95% CI, 93–100%] and specificity of 97% [95% CI, 91–100%]) (*p* < 0.01).

## 4. Discussion

We investigated the diagnostic performance of the MRPI for the differentiation of PSP from PD using the bivariate random-effects and HSROC models. Our updated meta-analysis demonstrated the excellent diagnostic performance of the MRPI in differentiating PSP from PD. The pooled sensitivity was 96% (95% CI, 87–99%), the pooled specificity was 98% (95% CI, 91–100%), and the area under the HSROC curve was 0.99 (95% CI, 0.98–1.00). Heterogeneity was present among the selected studies; however, meta-regression showed significantly higher sensitivity and specificity when using 3 T MRI compared with 1.5 T MRI. Therefore, the MRPI may have great potential to accurately differentiate PSP from PD and could help with the implementation of appropriate management strategies for patients with PSP.

Several studies have evaluated the diagnostic performance of the MRI for the differentiation of atypical parkinsonism from PD using various measurement methods and techniques, i.e., measurement of the midbrain area, pons area to midbrain area ratio, or MRPI and voxel-based morphometry using a supervised machine learning algorithm [13,43,49]. Our updated meta-analysis focused on 14 articles that used the MRPI only for the differentiation of PSP from PD. The main source of heterogeneity was the magnet field strength; however, the sensitivity and specificity of each subgroup were still high (all of the values were higher than 97%). Therefore, our study demonstrated that the MRPI could be used to differentiate PSP from PD.

The introduction of MRPI facilitated the differentiation of atypical parkinsonism from PD, but PSP-P was difficult to differentiate from PD with the MRPI [7,15]. MRPI 2.0 has been introduced to distinguish not only PSP-RS, but also PSP-P from PD, and several recent studies introduced MRPI 2.0 [19,23,58]. Notably, Quattrone et al. [19] reported that both MRPI and MRPI 2.0 had excellent diagnostic performances in differentiating PSP-RS from PD, but the MRPI 2.0 outperformed MRPI in distinguishing PSP-P from PD. As there were few studies on MRPI 2.0 [19,23,58], we studied on MRPI. If more research on MRPI 2.0 comes out, it will be necessary to analyze it. Furthermore, there have been attempts to differentiate atypical parkinsonism from PD using automated volumetry or machine learning algorithms [17,19,43,58]. Nigro et al. [17] demonstrated that automated measurement of the MRPI showed good performance in comparison with manual measurement. In addition, Salvatore et al. [43] suggested that a machine learning algorithm can allow the differentiation of PSP from PD. As described above, several notable studies using various measurement methods or techniques have been performed; however, subgroup analysis was not possible because of the paucity of the data. Further studies should be conducted to address the issue of paucity.

Although Zhang et al. [24] previously performed a systematic review and meta-analysis, there were several limitations in that study. First, their search strategy was inadequate; they searched for articles only from PubMed. In comparison, our study included articles from both PubMed and EMBASE. Second, Zhang et al. included articles that differentiated PSP patients from healthy controls; our study excluded 12 of 34 articles that did not differentiate PSP from PD, i.e., articles that differentiated PSP patients from non-PSP patients including multiple system atrophy or healthy controls. Our study focused on the differentiation of PSP from PD. Third, our study used the HSROC curve to evaluate heterogeneity and the accuracy of the MRPI, and Zhang et al. only used the summary receiver operating characteristic curve. Finally, their study did not perform meta-regression analysis. However, our study uncovered a major source of heterogeneity with meta-regression analysis.

There are several limitations in our meta-analysis. First, there was heterogeneity among the selected studies. We performed meta-regression analysis to address this problem. In addition, the potential source of the heterogeneity could be that the progression stages of PD and PSP differed between each study. Second, although several latest studies on the MRPI report results according to the PSP phenotype, we did not divide the PSP group into subgroups of patients with two major phenotypes. Further studies on the performance of the MRPI according to the PSP phenotype will be needed. Third, the age of the data was the limitation. The selected 14 articles included the studies published before 2017 that were based on old criteria of PSP diagnosis. Fourth, because the number of selected studies was small, we could not perform sub-group analysis. Last, slice thickness or whether 3D images were used may have affected our meta-regression which revealed higher sensitivity and specificity when using 3 T MRI compared with 1.5 T MRI. Further studies should be conducted with standardized patient groups and protocols.

In conclusion, our meta-analysis demonstrated that the MRPI may have great potential to accurately differentiate PSP from PD and could help with the implementation of appropriate management strategies for patients with PSP.

## Figures and Tables

**Table 1:** Study and patient characteristics of the selected articles.

Author (Year of Publication)	Institution	Duration of Patient Recruitment	No. of Patients (*n*)	PSP (*n*)	PD (*n*)	Mean Age of PD Patients (SD)	Mean Age of PD Patients (SD)	M:F (PSP)	M:F (PD)	Study Design	Consecutive Enrollment
Constantinides VC, et al., (2018)	National and Kapodistrian University of Athens	2011–2014	42	24	18	63.2 (6.8)	64.4 (9.3)	13:11	10:8	prospective	yes
Hussl A, et al., (2010)	Medical University Innsbruck	NA	97	22	75	68.7 (9.1)	64.8 (9.7)	11:11	46:29	NA	NA
Longoni G, et al., (2011)	University of Belgrade	1998.01–2008.11	35	10	25	62.5	65.5	3:7	6:19	retrospective	yes
Möller L, et al., (2017)	Five German academic centers (Universities in Marburg, Dusseldorf, Frankfurt, Freiburg, and Ulm)	2009–2013	310	106	204	69.0 (0.6)	64.0 (0.8)	60:46	136:68	retrospective	NA
Morelli M, et al., (2011)	NA	NA	340	42	298	70.26 (6.0)	NA	31:11	198:100	NA	NA
Nigro S, et al., (2019)	Seven different Italian movement disorder centers	NA	192	37	155	NA	NA	NA	NA	retrospective	NA
Nizamani WM, et al., (2017)	Khan University Hospital	2006.01–2015.12	68	34	34	66.8 (6.3)	66.8 (6.3)	19:15	20:14	retrospective	yes
Oktay C, et al., (2020)	Neurology Department, Movement Disorder Clinic	2015.11–2017.03	57	14	43	NA	NA	NA	NA	retrospective	NA
Picillo M, et al., (2020)	University of Salerno and University of Pisa	2015.11–2018.12	73	38	35	71	68	23:15	26:9	retrospective	yes
Quattrone A, et al., (2018)	University of Catanzaro	2009–2017	99	46	53	70.4 (5.2)	70.3 (5.2)	25:21	39:14	NA	yes
Quattrone A, et al., (2008)	NA	2002.06–2006.05	141	33	108	69.3 (6.1)	65.8 (9.0)	23:10	62:46	prospective	yes
Sankhla CS, et al., (2016)	P.D. Hinduja National Hospital	2012.03–2014.03	39	26	13	66.2 (7.4)	56.5 (11.2)	18:8	9:4	NA	yes
Sjöström H, et al., (2020)	Karolinska University Hospital	2001–2015	169	29	140	69.1 (6.7)	65.3 (9.8)	11:18	48:92	retrospective	yes
Zanigni S, et al., (2016)	NA	2010–2014	65	23	42	72.8 (7.1)	64.7 (10.5)	12:11	29:13	retrospective	yes

NA; not available, SD; standard deviation, PSP; progressive supranuclear palsy, PD; Parkinson’s disease, M; male, F; female.

**Table 2:** MRI characteristics of the selected articles.

Author (Year of Publication)	Magnet Strength (T)	Vendor	Scanner	Sequence	Section Thickness (mm)	Number of Readers	Experience of Readers	Measurement Method for the MRPI
Constantinides VC, et al., (2018)	1.5 or 3	Philips	NA	T1 or 3D T1 turbo field echo	1–5	1	NA	Manual
Hussl A, et al., (2010)	1.5	Siemens	Magnetom Symphony	Native 3D T1	1.2	NA	NA	Manual
Longoni G, et al., (2011)	1.5	Siemens	Magnetom Avanto	3D T1 MP-RAGE	NA	1	NA	Manual
Möller L, et al., (2017)	1.5 or 3	Siemens	Magnetom Trio	T1 3D MP-RAGE	1 or 1.2	2	NA	Manual
Morelli M, et al., (2011)	1.5	GE	Signa	T1 volumetric spoiled gradient echo	0.6	2	>10 yrs	Manual
Nigro S, et al., (2019)	3	GE	Discovery MR750, Signa HDx	3D T1	1–1.2	2	>8 yrs	Manual
Nizamani WM, et al. (2017)	1.5 or 3	Siemens	Avanto, Vantage	T1 volumetric spoiled gradient echo, T2, FLAIR	0.6, 4, 4	2	NA	Manual
Oktay C, et al., (2020)	3	Siemens	Spectra	3D T1 MP-RAGE	1	2	20/5 yrs	Manual
Picillo M, et al., (2020)	1.5 or 3	Siemens	Skyra	3D T1	NA	1	>15 yrs	Manual
Quattrone A, et al., (2018)	3	GE	MR750	3D T1 volumetric spoiled gradient echo	1	2	>10 yrs	Automatic
Quattrone A, et al., (2008)	1.5	GE	Signa	T1 volumetric spoiled gradient echo	0.6	2	NA	Manual
Sankhla CS, et al., (2016)	1.5 or 3	NA	NA	T1 volumetric spoiled gradient echo	NA	1	NA	Manual
Sjöström H, et al., (2020)	1.5 or 3	Siemens	Aera, Avanto, Symphony, Trio	3D T1 MP-RAGE	NA	2	NA	Automatic
Zanigni S, et al., (2016)	1.5	GE	Signa	3D volumetric T1—FSPGR	NA	3	NA	Manual

NA; not available, MRPI; magnetic resonance parkinsonism index, MP-RAGE; magnetization prepared rapid gradient echo, FLAIR; fluid-attenuated inversion recovery, FSPGR; fast spoiled gradient echo.

## Data Availability

Not applicable.
